# Perillaldehyde synergizes with ferroptosis inducers to promote ferroptotic cell death in gastric cancer

**DOI:** 10.3389/fcell.2025.1598520

**Published:** 2025-06-03

**Authors:** Rui Wang, Jin-Feng Cui, Jing Lv, Jia-Lin Song, Yang-Yang Lu, Xiao-Juan Huang, Zhong-Kun Lin, Si-Yi Zhang, Sha-Sha Wang, Wen-Sheng Qiu

**Affiliations:** ^1^ Department of Oncology, The Affiliated Hospital of Qingdao University, Qingdao, China; ^2^ Department of Oncology, Shandong Provincial Third Hospital, Jinan, Shandong, China

**Keywords:** natural products, perillaldehyde, gastric cancer, oxidative stress, ferroptosis

## Abstract

**Introduction:**

As a traditional medicine and food homologous plant, Perilla frutescens is widely cultivated in China, Japan, and Korea. According to the *Compendium of Materia Medica*, the leaves, stems, and seeds of perilla can all be used as medicine. Perilla essential oil has been used in traditional Chinese medicine since ancient times. It has been demonstrated that perillaldehyde (PAH), a primary composition of the essential oil extracted from perilla, can inhibit tumor growth through multiple mechanisms. However, the specific mechanisms by which PAH suppresses gastric cancer remain incompletely understood.

**Methods:**

We performed *in vitro* experiments using three cell lines (AGS, HGC27, and MFC) to assess the effects of PAH on cell viability, proliferation, and migration of gastric cancer cells. Concurrently, we established a subcutaneous gastric tumor model in BALB/c nude mice for *in vivo* animal studies. Subsequently, oxidative stress was measured via fluorescence staining techniques (H_2_DCFDA, DHE, and JC-1). We then evaluated whether PAH induced ferroptosis in gastric cancer cells through FerroOrange staining, quantification of intracellular glutathione (GSH) and lipid peroxidation levels, and Western blotting. Finally, PAH was co-administered with the ferroptosis inhibitor Ferrostatin-1 (Fer-1) or the ferroptosis inducer RSL3, and relevant experiments were re-evaluated.

**Results:**

In this study, PAH was proven to inhibit the growth of gastric cancer both *in vivo* and *in vitro*. It led to a reduction in mitochondrial membrane potential (MMP), an augmentation of the accumulation of reactive oxygen species (ROS), and an elevation of oxidative stress levels. Moreover, PAH decreased intracellular GSH levels while increasing intracellular lipid peroxidation and Fe^2+^ levels. These effects indicate that PAH induces ferroptosis via inhibiting the system Xc (−)/GSH/GPX4 axis. Furthermore, PAH influenced the expression of proteins related to iron transport and storage and regulated ferroptosis via the P62-Keap1-Nrf2 pathway. When combined with the ferroptosis inducer RSL3, PAH could promote ferroptosis in gastric cancer.

**Discussion:**

Our research suggests a potential therapeutic strategy in which PAH could be used to synergize with ferroptosis inducers for treating gastric cancer.

## 1 Introduction

Gastric cancer stands as one of the most widespread malignant tumors globally and remains a predominant contributor to cancer-related mortality worldwide (Guan et al., 2023). Based on the most recent global cancer statistics, gastric cancer holds the fifth position in terms of incidence (4.9%) and mortality (6.8%) (Bray et al., 2022). Regrettably, the majority of patients are diagnosed at an advanced stage as a consequence of the absence of specific early symptoms and the low prevalence of routine screening (Zhou et al., 2023). At present, systemic chemotherapy continues to serve as the fundamental and principal approach in the treatment of advanced gastric cancer. However, the complex molecular mechanisms underlying the disease often result in resistance to chemotherapy drugs, contributing to poor patient outcomes (Gorrini et al., 2013). Besides, these chemotherapy drugs are frequently associated with significant adverse reactions and toxic side effects. Given the high mortality rate and the challenges posed by the side effects of existing treatments, there exists an immediate and pressing necessity to devise novel therapeutic agents and groundbreaking treatment strategies so as to improve patient prognosis.

The regulation of redox equilibrium is crucial for guaranteeing the normal survival of cells and preserving cellular functionality (Gorrini et al., 2013). The equilibrium between the generation of reactive species and the activation of antioxidant mechanisms determines the redox status of cells. ROS are highly reactive molecules that exert an important influence on the normal physiological functions of cells and the processes of signal transduction (Dodson et al., 2019). As byproducts of normal cellular metabolism, ROS influence intracellular oxidative stress levels (Reuter et al., 2010). In cancer cells, elevated levels of aerobic glycolysis and oxidative stress are hallmark features (Cairns et al., 2011), and the initiation and progression of cancer are intricately linked to oxidative stress (Visconti and Grieco, 2009). ROS exhibits a dual role in tumor progression, functioning simultaneously as a promoter and a suppressor of cancer progression. This duality reflects the context-dependent nature of ROS, which varies according to the cellular environment, tissue type, and stage of cancer (Sarmiento-Salinas et al., 2021). At low to moderate concentrations, ROS can facilitate tumorigenesis by acting as signaling molecules or inducing genomic DNA mutations. Conversely, high ROS could cause cellular damage and trigger cell death, particularly during the early stages of tumor development (Gorrini et al., 2013). During this phase, the intracellular antioxidant response system is also activated to counteract oxidative stress. The transcription factor, nuclear factor erythroid 2-related factor 2 (Nrf2), serves as a core regulator of the cellular antioxidant reactions and assumes a crucial function in adjusting the oxidative stress within tumor cells (Dodson et al., 2019). Kelch-like ECH-associated protein 1 (Keap1) exerts a negative regulatory effect on Nrf2 (Hayes and McMahon, 2009). Sequestosome 1 (SQSTM-1), also recognized as P62, is an endogenous protein that induces Nrf2 activation (Komatsu et al., 2010). Under stress conditions, the expression of P62 is increased. As a reaction to oxidative stress, P62 facilitates the aggregation of Keap1 in a non-canonical manner through its protein sequestration function (Wang L. et al., 2023). Upon the interaction between Keap1 and P62, Keap1 becomes sequestered within the protein aggregates mediated by P62 (Wang et al., 2021), resulting in reduced Nrf2 ubiquitination, inhibition of its degradation, and increased stability, thereby activating the transcription of Antioxidant response element (ARE) and its downstream target genes (Lau et al., 2010). Thus, P62 can regulate oxidative stress levels in tumor cells via modulating the Keap1-Nrf2 pathway. Similar to the dual role of ROS in tumor cells, the P62-Keap1-Nrf2 also exhibits contradictory effects in cancer. On one hand, the expression of P62 stimulates the proliferation of bladder cancer cells via the activation of the Keap1-Nrf2 pathway (Li et al., 2020), and its upregulation in the early stages of tumorigenesis can induce hepatocellular carcinoma (Umemura et al., 2016). On the other hand, elevated levels of P62 within hepatic stellate cells have been demonstrated to impede the progression of hepatocellular carcinoma (Duran et al., 2016). TRIM21-mediated ubiquitination of P62 can suppress the P62-Keap1-Nrf2 pathway, thereby promoting liver cancer (Wang et al., 2021). These discoveries imply that the P62-Keap1-Nrf2 pathway may exert distinct and context-dependent roles at different stages of tumor development.

Ferroptosis, a type of regulated cell death (RCD) (Tang et al., 2019), is characterized by the buildup of iron-dependent lipid peroxidation products and ROS (Dixon et al., 2012). The initiation of ferroptosis is intricately associated with the existence of iron ions, particularly unstable Fe^2+^, which significantly elevates the risk of oxidative stress. This occurs through the Fenton reaction, leading to the generation of hydroxyl radicals and subsequent oxidative damage, especially to cell membranes via lipid peroxidation (Li et al., 2024). The glutathione peroxidase 4 (GPX4) regulatory pathway represents the classic mechanism of ferroptosis. As a crucial modulator of this pathway, GPX4 serves as the unique enzyme responsible for reducing lipid peroxides (Zhang et al., 2025). Depletion of GPX4 leads to accumulation of peroxidized lipids, thereby inducing ferroptosis. Conversely, upregulation of GPX4 expression suppresses lipid peroxidation and consequently inhibits ferroptosis. Extensive research has indicated that ferroptosis plays a key role in inhibiting the occurrence and development of tumors and is a new opportunity for cancer treatment (Zhang et al., 2022).

Natural products have shown enormous potential in inducing ferroptosis in tumor cells. PAH, which serves as the principal constituent of the essential oil derived from the Perilla plant (Masumoto et al., 2019), is a monocyclic terpenoid compound known for its diverse biological activities (Zielińska-Błajet et al., 2021), encompassing antifungal (Chen et al., 2020; Chu et al., 2024), anti-inflammatory (Fan et al., 2020), antioxidant (Fuyuno et al., 2018), antidepressant (Song et al., 2018), and antitumor properties (Zielińska-Błajet et al., 2021). In recent years, PAH has exhibited anti-tumor activity against diverse cancer cell lines, such as human squamous cell carcinoma of the tongue (BroTo), human lung adenocarcinoma (A549) (Elegbede et al., 2003), mouse gastric cancer cells (MFC) (Zhang et al., 2019), and human prostate cancer cells (PC-3) *in vitro* (Lin et al., 2022). Studies have shown that PAH may exert a significant influence on the process of ferroptosis. For instance, PAH has been proven to mitigate ionizing radiation-induced ferroptosis in intestinal crypt cells via the Nrf2 signaling pathway, thereby alleviating intestinal damage (Tang et al., 2023). In addition, PAH has been characterized as a novel ferroptosis inducer, capable of promoting ferroptosis in two leukemia cell lines, highlighting its potential in treating acute myeloid leukemia (Catanzaro et al., 2022). Nevertheless, it is still not clear if PAH is capable of inducing ferroptosis in gastric cancer cells.

## 2 Materials and methods

### 2.1 Reagents and antibodies

Perillaldehyde (PAH, S3205, Selleck), dimethyl sulfoxide (DMSO, HY-Y0320, MCE), Z-VAD-FMK (S7023, Selleck), Necrostatin-1 (Nec-1; S8037, Selleck), Ferrostatin-1 (Fer-1; HY-100579, MCE), (1S, 3R)-RSL3 (RSL3; Y-100218A, MCE), Doxorubicin (DOX; T1020, TargetMol), Cisplatin (CIS; S1166, Selleck), 3-(4,5-dimethylthiazol-2-yl)-2,5-diphenyltetrazolium bromide (MTT; M158055, Aladdin), crystal violet (C110703, Aladdin), 5-Fluorouracil (5-FU; HY-90006, MCE), 2′,7′-dichlorodihydrofluorescein diacetate (H_2_DCFDA; DCF; HY-D0940, MCE), dihydroethidium (DHE; 50102ES02, Yeasen Biotechnology), JC-1 mitochondrial membrane potential assay kit (JC-1; J6004S, UE Landy Biotechnology), Calcein/PI cell viability and cytotoxicity assay kit (C2015M, Beyotime Biotechnology), Hieff Trans® *in vitro* siRNA/miRNA Transfection Reagent (40806ES02, Yeasen Biotechnology), GSH and GSSG assay kit (S0053, Beyotime Biotechnology), divalent iron ion detection probe (FerroOrange; F374, Dojindo), and lipid peroxidation sensor (BODIPY 581/591 C11; D3861, Invitrogen). Recombinant glutathione peroxidase 4 (GPX4) rabbit monoclonal antibody (ab125066, Abcam), solute carrier family 7 member 11 (SLC7A11; xCT) rabbit polyclonal antibody (26864-1-AP, Proteintech), ferritin heavy chain (FTH1) rabbit polyclonal antibody (11682-1-AP, Proteintech), ferritin light chain (FTL) rabbit polyclonal antibody (10727-1-AP, Proteintech), GAPDH mouse monoclonal antibody (60004-1-Ig, Proteintech), SQSTM1/P62 (D1Q5S) rabbit monoclonal antibody (#39749, Cell Signaling Technology), Nrf2 rabbit monoclonal antibody (ab62352, Abcam), Nrf2 rabbit polyclonal antibody (R1312-8,HUABIO), Goat Anti-Rabbit IgG (H + L) (Elab Fluor® 488 conjugated) (E-AB-1055,Elabscience), Keap1 rabbit monoclonal antibody (ab227828, Abcam), NAD(P)H quinone dehydrogenase 1 (NQO1) rabbit monoclonal antibody (ab80588, Abcam), and heme oxygenase-1 (HO-1/HMOX1) rabbit monoclonal antibody (ab189491, Abcam).

### 2.2 Cell culture

AGS and HGC27 cell lines were procured from the Cell Bank of Type Culture Collection of the Chinese Academy of Sciences located in Shanghai, while the MFC cell line was acquired from Wuhan Pricella Life Science & Technology Co., Ltd. All three cell lines underwent authentication via short tandem repeat (STR) profiling, and it was confirmed that they were devoid of *mycoplasma* contamination. These cells were cultivated in Roswell Park Memorial Institute (RPMI)-1640 medium (Pricella Life Science & Technology Co., Ltd., China), and the medium was supplemented with 10% fetal bovine serum (FBS; Life-iLab, China), along with 100 U/mL penicillin and 100 μg/mL streptomycin (Pricella, Wuhan, China). All cells were incubated at a temperature of 37°C within a humidified environment containing 5% CO_2_. The culture medium was replaced every 2 days.

### 2.3 MTT cell viability assay

After subjecting the cells to treatment with PAH, either alone or together with Fer-1 and RSL3, we used the MTT colorimetric assay to evaluate cell viability. In brief, AGS and HGC27 cells were evenly plated in 24-well plates and allowed to stabilize in an incubator 24 h prior to treatment. Following the treatment period, the culture medium was carefully aspirated. Subsequently, the cells were incubated with an MTT assay working solution (0.5 mg/mL) for 2–4 h in the incubator. After incubation, the supernatant was discarded, and the formed formazan crystals were solubilized in DMSO. The absorbance was then determined at a wavelength of 490 nm using a microplate reader (PerkinElmer, United Kingdom).

### 2.4 Colony formation assay

AGS and HGC27 cells were collected and seeded into 6-well plates at a density of 1,000 cells per well. After the cells had firmly attached to the plate surface, they were exposed to PAH either alone or in combination with Fer-1 and RSL3. Subsequently, the cells were cultivated for a period ranging from 10 to 14 days. When colonies became visible (each containing more than 50 cells), the culture process was halted. The cells were washed twice with phosphate-buffered saline (PBS), fixed with 4% paraformaldehyde, and stained with 0.1% crystal violet for 60 min. The quantification of colonies formed was carried out using ImageJ software.

### 2.5 Wound healing assay

Gastric cancer cells were inoculated into 6-well plates and incubated for 24 h to reach a stable state. A sterile 100 µL pipette tip was then used to generate a scratch across the cell monolayer. The dislodged cells were gently rinsed off with PBS. Images of the scratch were captured at 0 and 24 h using an inverted microscope under bright field conditions. The extent of cell migration was subsequently quantified and analyzed using ImageJ software.

### 2.6 Cell viability and cytotoxicity assay

The Calcein AM/PI cell viability and cytotoxicity assay kit (C2015S, Beyotime) was utilized to assess cell viability subsequent to treatment with PAH alone or in combination with Fer-1 or RSL3. The working solution, prepared according to the kit instructions, was added to the cells, which were then incubated in a CO_2_ incubator at 37°C in the dark for 30 min. After the incubation period, the staining results were inspected using an inverted fluorescence microscope to assess the treatment effects.

### 2.7 Animal experiments

Beijing Vital River Laboratory Animal Technology Co., Ltd. supplied female 4-week-old BALB/c nude mice, which were housed in the Experimental Animal Center of the Qingdao University Biomedical Center. Prior to the experiment, the mice were subjected to a 1-week acclimation phase within a specific pathogen-free environment where temperature, humidity, and lighting conditions were meticulously controlled. All experimental procedures were conducted in strict accordance with the Guidelines for the Ethical Treatment of Experimental Animals and received approval from the Ethics Committee of the Affiliated Hospital of Qingdao University (NQ: 20241025BALB/c-nu3220241125193). MFC cells (1.5 × 10^5^) were administered via subcutaneous injection into the dorsal area of each mouse. Then, the mice were allocated into four groups randomly: a control group, a 5-FU group (20 mg/kg), a low-dose PAH group (50 mg/kg), and a high-dose PAH group (100 mg/kg), with four mice in each group. PAH was administered via intragastric perfusion, while 5-FU was delivered through intraperitoneal injection, with treatments administered every 2 days. Body weight and tumor volume (mm^3^) calculated as length × width^2^ × 0.5 were recorded before each treatment. Two weeks after the formation of subcutaneous tumors, the mice were euthanized, and tumor tissues were collected, weighed, photographed, and measured. Besides, the hearts, livers, spleens, and kidneys were harvested, immobilized in paraformaldehyde, and sent to Wuhan Servicebio Biotechnology Co., Ltd. for histopathological examination.

### 2.8 MMP assay

The JC-1 Mitochondrial Membrane Potential Assay Kit (JC-1; J6004S, UE Landy Biotechnology) was utilized to prepare the JC-1 staining working solution. After experimental treatment, the cells were incubated with the staining solution at 37°C in a cell culture incubator for 30 min. The MMP changes were visualized and analyzed using an inverted fluorescence microscope (Nikon, Japan).

### 2.9 ROS assay

HGC27 and AGS cells were seeded in 24-well plates and stabilized in an incubator for 24 h prior to treatment. Following the treatment, 10 μmol/L DCF and DHE working solutions, prepared in FBS-free RPMI-1640 medium, were added to the cells. Subsequently, the cells were incubated at 37°C in a dark environment for 30 min. After incubation, the cells were washed 2–3 times, and ROS levels were detected under an inverted fluorescence microscope. Semi-quantitative analysis of the ROS levels was subsequently conducted using ImageJ software.

### 2.10 RNA sequencing and differential gene enrichment analysis

AGS cells were lysed TRIzol Reagent (Sevicebio Biotechnology Co., Ltd., Wuhan, China) after a 24-h incubation with 200 μmol/L PAH to extract total RNA. The quality of the RNA samples was assessed using a NanoDrop™ One/OneC Microvolume UV-Vis Spectrophotometer (Thermo Fisher, United States), the Qubit™ RNA HS Assay Kit (Thermo Fisher, United States), and the Agilent 4200 TapeStation system (Agilent Technologies, CA, United States). RNA sequencing was subsequently conducted by Jinghong Biotechnology Co., Ltd. (Jinan, China), and the clusterProfiler hypergeometric distribution algorithm was applied to perform pathway enrichment analysis of differentially expressed genes.

### 2.11 Immunofluorescence analysis

AGS and HGC27 cells were seeded in 10 mm glass-bottom culture dishes and incubated overnight. The next day, the cells were treated with PAH for 24 h. Subsequently, they were fixed with 4% paraformaldehyde and permeabilized with 0.2% Triton X-100, followed by blocking with 5% bovine serum albumin (BSA). The cells were then incubated overnight with a primary antibody against Nrf2 (R1312-8; HUABIO), followed by incubation with a fluorescent secondary antibody (E-AB-1005; Elabscience). Finally, cell nuclei were counterstained with DAPI, and images were captured using a confocal laser scanning microscope (Leica, Germany).

### 2.12 Intracellular Fe^2+^ assay

HGC27 and AGS cells were seeded at a density of 3 × 10^5^ cells/well in 6-well plates and incubated overnight at 37°C in a 5% CO_2_ incubator. After the experimental treatment, a FerroOrange working solution with a concentration of 1 μmol/L was added to the cells, and the cells were incubated at 37°C in the dark for 30 min. Subsequently, the cells were promptly examined under a fluorescence microscope to assess intracellular Fe^2+^ levels, and semi-quantitative analysis was conducted using ImageJ software to evaluate the results.

### 2.13 Intracellular GSH level assay

In accordance with the instructions provided by the manufacturer of the GSH and GSSG Assay Kit (S0053, Beyotime), AGS and HGC27 cells were subjected to treatment. The absorbance was then measured at 412 nm using a microplate reader (PerkinElmer, United Kingdom) to determine the intracellular GSH content.

### 2.14 BODIPY-581/591 C11 lipid peroxidation assay

HGC27 and AGS cells were plated in 6-well plates at a seeding density of 3 × 10^5^ cells per well. On the following day, the cells were treated with PAH either alone or in combination with Fer-1 and RSL3 for an additional 24 h. A 10 μmol/L working solution of BODIPY 581/591 C11, which had been diluted in FBS-free RPMI-1640 medium, was introduced to the treated cells. Subsequently, the cells were incubated in the dark for 30 min. Upon completion of the incubation, cells were digested with 0.25% trypsin, washed twice with PBS, then removed the supernatant, and cells were resuspended with PBS. The level of intracellular lipid peroxidation was then analyzed by means of a flow cytometer (Beckman, United States).

### 2.15 Western blotting

HGC27 and AGS cells were counted and seeded into cell culture dishes. Following treatment, the cells were gently collected using a cell scraper and lysed on ice for 1 h using Western and IP cell lysis buffers (P0013, Beyotime) supplemented with PMSF and phosphatase inhibitors. The lysate was centrifuged, and the protein concentration in the supernatant was measured using a BCA protein assay kit (Elabscience, Wuhan). The samples were then mixed with 5X loading buffer (LT101S, Elabscience) and denatured by boiling at 100°C. Electrophoresis was performed at 200 V for 35 min using a one-step PAGE gel rapid preparation kit (PG212, Elabscience), after which the proteins were transferred to a polyvinylidene fluoride (PVDF) membrane (10600023, Cytiva) at 300 mA for 2 h. The membrane was blocked with 5% skim milk for 2 h and subsequently incubated with primary antibodies (GPX4, SLC7A11, TFRC, FTL, FTH1, P62, Nrf2, Keap1, NQO1, HO-1/HMOX1, GAPDH) overnight at 4°C. The membrane was then incubated with HRP-conjugated secondary antibodies for 2 h with gentle shaking. Protein bands were visualized using an ECL chemiluminescent solution and quantified using ImageJ software.

### 2.16 Small interfering RNA (siRNA) transfection

Nrf2 siRNA was synthesized by Shanghai GenePharma Pharmaceutical Technology Co., The sequences used were as follows:

siNrf2–1 target sequence: 5′–CAGUCUUCAUUGCUACUAAUCTTGAUUAGUAGCAAUGAAGACUGTT–3′; siNrf2–2 target sequence: 5′–GACAGAAGUUGACAAUUAUCATTUGAUAAUUGUCAACUUCUGUCTT–3′; siNrf2–3 target sequence: 5′–CAUUGAUGUUUCUGAUCUAUCTTGAUAGAUCAGAAACAUCAAUGTT–3′; negative control target sequence: 5′–UUCUCCGAACGUGUCACGUTTACGUGACACGUUCGGAGAATT–3′; HGC27 and AGS cells were plated in 24-well plates at a seeding density of 4 × 10^3^ cells per well. After 24 h of cell culture in the incubator, Hieff Trans ® *in vitro* siRNA/miRNA Transfection Reagent (40806ES02; Yeasen Biotechnology) was used to transfect siRNA into cells. After transfection, cells were incubated in the culture incubator for 6–8 h, followed by medium replacement. The cells were then cultured for an additional 72 h before subsequent processing.

### 2.17 Statistical analysis

Each experimental result was derived from three independent replicates (*n* = 3). All the statistical data were presented in the form of mean ± standard deviation (SD). Statistical analyses were carried out utilizing GraphPad Prism 9.5.1 software. One-way analysis of variance (ANOVA) was initially conducted, followed by multiple comparison tests to examine the disparities among groups. A *p-*value < 0.05 was regarded as statistically significant, with specific thresholds denoted as follows: no statistical significance (ns) for *p* > 0.05, * for *p* < 0.05, ** for *p* < 0.01, *** for *p* < 0.001, and **** for *p* < 0.0001. Flow cytometry data were analyzed and visualized using FlowJo software (Version 10.8.1).

## 3 Results

### 3.1 PAH inhibits the growth of gastric cancer cells *in vitro* and *in vivo*

**FIGURE 1:**
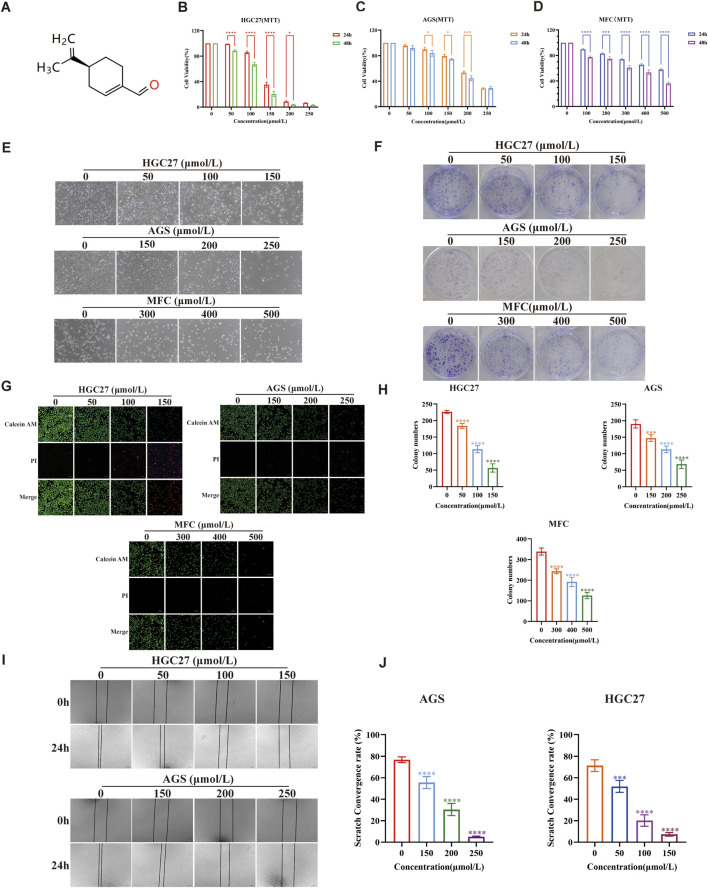
PAH inhibits the growth of gastric cancer cells *in vitro* **(A)** Chemical structure of perillaldehyde. **(B,C)** Human gastric cancer cell lines HGC27 and AGS were treated with the indicated concentrations of PAH for 24 or 48 h. Cell viability was assessed by MTT assay (*n* = 4). **(D)** Mouse gastric cancer cell lines MFC were treated with different concentrations of PAH for 24 or 48 h. Cell viability was assessed by MTT assay (*n* = 4). **(E)** Changes in cell morphology after PAH treatment of HGC27 and AGS cells. Scale bar, 100 μm. **(F,H)** Cell colony formation of HGC27, AGS and MFC cells treated with different concentrations of PAH, and the number of cells forming colonies was quantified using ImageJ software. **(G)** Calcein AM/PI fluorescence staining of HGC27, AGS and MFC cells treated with different concentrations of PAH, Calcein AM staining of live cells, showing green fluorescence; (PI) staining of dead cells, showing red fluorescence. Scale bar, 100 μm. **(I,J)** Cell migration of HGC27 and AGS cells treated with different concentrations of PAH for 24 h by wound healing assay, and the cell migration area was quantified by ImageJ to calculate the cell migration rate. Scale bar, 100 μm. (Data are expressed as mean ± SD, *n* = 4. ns, not statistically significant, * indicates compared with the control group, *p < 0.05, **p < 0.01, ***p < 0.001, ****p < 0.0001).

Figure 1A illustrates a schematic diagram of the PAH structure. To evaluate the influence of PAH on cell viability, the MTT colorimetric assay was employed in HGC27, AGS, and MFC. Based on previous research findings regarding PAH’s effects on other tumor cell lines, we chose concentrations within the range of 50–500 μmol/L and treated the cells for 24 h or 48 h to assess viability. PAH treatment for 24 h caused an obvious decrease in cell viability across all three gastric cancer cell lines, exhibiting a dose-dependent effect. Moreover, after 48 h of PAH treatment, cell viability decreased even further compared to the 24-h treatment (Figures 1B–D). We next determined the IC50 values using Prism software, which revealed that the three cell lines treated with PAH for 24 h had IC50 values of 135.3 μmol/L, 204.2 μmol/L, and 664.2 μmol/L, respectively. For the 48-h treatment, the IC50 values were 114.0 μmol/L, 191.6 μmol/L, and 394.1 μmol/L. Following PAH treatment and fixation with 4% paraformaldehyde, we examined the morphological changes and the number of surviving HGC27, AGS, and MFC cells under a microscope. With increased PAH concentration and treatment duration, the number of surviving cells gradually decreased, demonstrating a time- and concentration-dependent relationship between drug exposure and cell viability. The cells exhibited irregular, spherical, and shiny morphologies, along with reduced adhesion capacity, while maintaining normal nuclear morphology (Figure 1E). We selected 24 h as the treatment time point for HGC27 and AGS cells based on the calculated IC50 values. The integer value closest to the IC50 at the corresponding treatment time or each cell line was chosen as the midpoint of the drug concentration gradient. Subsequently, two drug concentration groups within a range of 50 μmol/L on either side of this midpoint were selected for further experiments. For MFC cells, 48 h was chosen as the treatment time point, and the integer value closest to the IC50 after 48 h of PAH treatment was used as the midpoint of the drug concentration gradient. Two drug concentration groups within a range of 100 μmol/L on either side of this midpoint were selected for subsequent experiments. HGC27 cells were treated with 50 μmol/L, 100 μmol/L, and 150 μmol/L PAH for 24 h; AGS cells were treated with 150 μmol/L, 200 μmol/L, and 250 μmol/L PAH for 24 h; and MFC cells were treated with 300 μmol/L, 400 μmol/L, and 500 μmol/L PAH for 48 h (Figure 1G). Cell survival was evaluated using Calcein-AM/PI fluorescence staining. We found that with the drug concentration increased, the green fluorescence of the cells weakened while the red fluorescence intensified, suggesting that PAH inhibits the proliferation of HGC27, AGS, and MFC gastric cancer cells. PAH treatment was found to reduce the formation of cell colonies (Figure 1F). The colony formation rate was quantified using ImageJ software (Figure 1H), revealing that PAH exhibited significant antiproliferative effects on HGC27, AGS, and MFC cells in a concentration-dependent manner. Given the observed differences in the cytotoxic activity of PAH against the three cell lines, we employed the cell scratch assay to further investigate its effects on cell migration. HGC27 and AGS, two human gastric cancer cell lines that demonstrated greater sensitivity to PAH, were selected for this analysis. These cells were treated with PAH for 24 h (Figure 1I), and the cell migration rate post-scratching was calculated using ImageJ software (Figure 1J). The results indicated that higher PAH concentrations led to a gradual reduction in the migration area and a decreased migration rate in both HGC27 and AGS cells.

**FIGURE 2:**
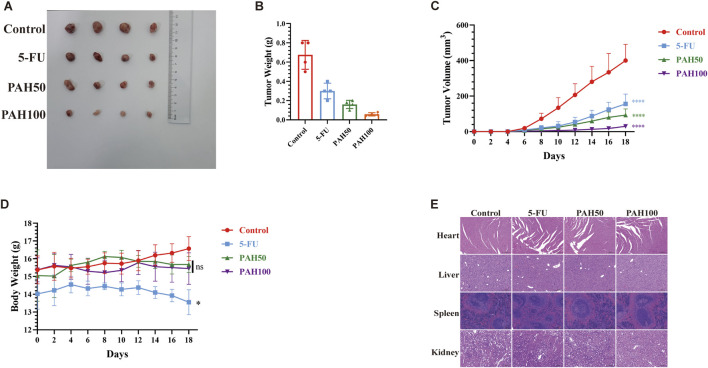
PAH inhibits the growth of gastric cancer cells *in vivo* **(A)** Tumor images and tumor weight **(B)**, tumor volume **(C)** and body weight changes **(D)** of MFC cell subcutaneous tumors in BALB/c nude mice (*n* = 4). **(E)** Representative images of the heart, liver, spleen and kidney of the MFC cell BALB/c nude mouse model treated with different groups (HE staining). Scale bar: 200 μm. (Data are expressed as mean ± SD, *n* = 4. ns, not statistically significant, * indicates compared with the control group, *p < 0.05, **p < 0.01, ***p < 0.001, ****p < 0.0001).

To assess the efficacy of PAH in inhibiting the two tumor cells *in vivo*, we utilized MFC cells, which exhibit low sensitivity to PAH, to further validate its anti-tumor activity. MFC cells were subcutaneously injected into BALB/c nude mice to establish a tumor model, and the mice were divided into four groups randomly: a control group, a positive control group (5-FU, 20 mg/kg), a low-concentration PAH treatment group (50 mg/kg), and a high-concentration PAH treatment group (100 mg/kg). After 2 weeks of PAH treatment, both PAH treatment groups and the 5-FU group exhibited significantly reduced tumor weight and volume, with PAH demonstrating superior inhibitory effects on tumor growth (Figures 2A–C). We monitored mouse weight and conducted histopathological analysis of major organs using hematoxylin-eosin (HE) staining. Results showed no significant weight changes or notable pathological alterations in the heart, liver, spleen, or kidneys of PAH-treated mice (Figures 2D,E), whereas the 5-FU group experienced weight loss. These findings indicate that PAH could effectively inhibit gastric cancer tumor growth *in vivo* with a high safety profile, causing no significant toxic side effects.

### 3.2 PAH increases the level of oxidative stress in gastric cancer cells

**FIGURE 3:**
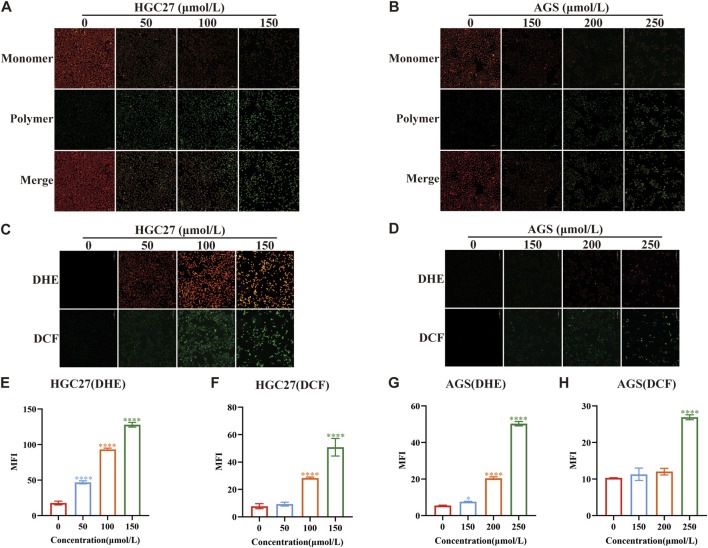
PAH increases the level of oxidative stress in gastric cancer cells **(A,B)** JC-1 dye was used to detect changes in mitochondrial membrane potential in HGC27 and AGS cells after PAH treatment for 24 h (*n* = 4). Scale bar: 100 μm. **(C,D)** Dihydroethidium (DHE) and H_2_DCFDA (DCF) fluorescence staining were used to evaluate the ROS levels in HGC27 and AGS cells after PAH treatment for 24 h, DHE (red fluorescence), DCF (green fluorescence). Scale bar: 100 μm. **(E–H)** ImageJ software was used to conduct semi-quantitative analysis of DHE and DCF fluorescence images of HGC27 and AGS cells for mean fluorescence intensity (MFI) (*n* = 4). (Data are expressed as mean ± SD; *p < 0.05, **p < 0.01, ***p < 0.001, ****p < 0.0001, significantly different from the control group).

ROS represent a group of unstable oxygen-containing compounds, including free radicals and non-radical molecules, primarily generated as byproducts of mitochondrial metabolic reactions. Consequently, ROS production within cells primarily stems from mitochondria (Gorrini et al., 2013), and an elevation in ROS levels contributes to increased oxidative stress. Previous studies on perillaldehyde have convincingly demonstrated its role in regulating oxidative stress (Fuyuno et al., 2018). Therefore, to investigate whether the toxicity of PAH to gastric cancer cells and their inhibitory effects on gastric cancer growth are linked to heightened oxidative stress, we assessed MMP as an indicator of mitochondrial integrity. Using HGC27 and AGS, which are sensitive to PAH, we measured MMP with JC-1 staining (Figures 3A,B). The results demonstrated that as PAH concentrations increased, the number of JC-1 monomers rose, while JC-1 polymers declined, indicating a significant reduction in MMP and mitochondrial depolarization. To quantify ROS levels, we employed fluorescent dyes DHE and H_2_DCFHDA (DCF) in HGC27 and AGS cells (Figures 3C,D), followed by a semi-quantitative analysis of fluorescence intensity (Figures 3E–H). The data demonstrated a dose-dependent increase in both green (DCF) and red (DHE) fluorescence intensities, confirming that PAH elevates ROS levels in these gastric cancer cell lines.

### 3.3 PAH induces oxidative stress while activating the P62-Keap1-Nrf2 pathway

**FIGURE 4:**
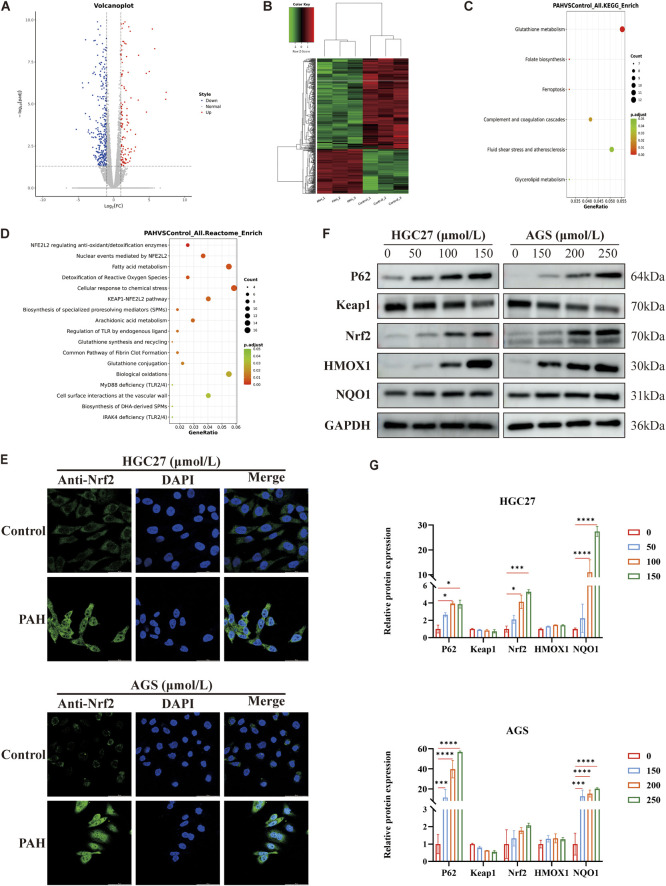
PAH induces oxidative stress while activating the P62-Keap1-Nrf2 pathway **(A)** AGS cells were treated with 200 μmol/L PAH for 24 h, and RNA sequencing volcano plot showed significantly differentially expressed genes. Differential genes are screened according to the threshold set by | log2(FoldChange) | and *p*-adj (<0.05). Blue indicates genes with downregulated expression, and red indicates genes with upregulated expression. **(B)** Inter-sample clustering heatmap for RNA sequencing. Green indicates low gene expression, red indicates high gene expression, and the connecting lines represent clustering results. **(C)** KEGG functional enrichment analysis of AGS cells treated with PAH, the results with *p*.adjust <0.05 and the top 20 gene numbers are selected for display. The size of the dots indicates the number of differential genes enriched in this pathway, the redder the dot color is, the more significant it is. **(D)** Compared with the control group, the results of Reactome pathway enrichment analysis of differential genes in AGS cells treated with PAH. The redder the dot color, the more significant it is. **(E)** AGS and HGC27 cells were treated with different concentrations of PAH for 24 h. Measurement of Nrf2 protein nuclear localization by laser confocal microscopy. Scale bar: 50 μm. **(F,G)** HGC27 and AGS cells were treated with corresponding concentration gradient PAH for 24 h. Western blotting was used to detect the protein expression of P62, Keap1, Nrf2, HMOX1 and NQO1 (*n* = 3) and ImageJ software was used to quantify the protein band (Data are expressed as mean ± SD; *p < 0.05, **p < 0.01, ***p < 0.001, ****p < 0.0001, significantly different from the control group).

We conducted RNA sequencing on AGS cells treated with 200 μmol/L PAH, with each group replicated four times. The gene expression profiles showed that PAH modulates gene translation in AGS cells. In the volcano plot and heatmap, blue and red represent downregulated and upregulated genes, respectively. The analysis demonstrated that PAH treatment resulted in 508 differentially expressed genes (DEGs), comprising 166 upregulated and 342 downregulated genes (Figures 4A,B). The predominance of downregulated genes suggests that PAH significantly suppresses gene transcription in AGS cells, leading to altered cellular functions. It was revealed by KEGG functional enrichment analysis that DEGs were chiefly associated with glutathione metabolism (Figure 4C). Reactome pathway enrichment analysis highlighted significant differences in pathways related to *NRF2* antioxidant regulation, *NRF2*-mediated nuclear events, the *KEAP1-NRF2* pathway, ROS detoxification, and fatty acid metabolism (Figure 4D). Under normal circumstances, the ubiquitin-proteasome pathway targets Nrf2 for degradation, with Keap1 acts as an adaptor protein for the ubiquitin ligase complex which is responsible for this process (Taguchi et al., 2011). However, when tumor cells are exposed to elevated levels of ROS, Keap1 becomes inactivated. This inactivation stabilizes intracellular Nrf2 levels, allowing Nrf2 to translocate to the nucleus. There, it forms heterodimers with small Maf proteins and binds to the ARE (Ichimura et al., 2013), activating the transcription of downstream antioxidant genes such as *heme oxygenase-1 (HO-1/HMOX1)*, *NAD(P)H quinone oxidoreductase 1 (NQO1)*, *solute carrier family 7 member 11 (SLC7A11; xCT)*, *glutathione peroxidase 4 (GPX4)*, *ferritin heavy chain (FTH1)* (Chang et al., 2018). Given that Nrf2 is a critical regulator of the P62-Keap1-Nrf2 pathway, and to determine whether PAH induces oxidative stress while activating the P62-Keap1-Nrf2 pathway, we first employed immunofluorescence to examine the subcellular localization of Nrf2 following PAH treatment. This approach allowed us to verify whether PAH potentially activates Nrf2 and promotes its nuclear translocation. The results demonstrated that after 24 h of PAH treatment, the fluorescence intensity of Nrf2 protein in the nucleus was significantly enhanced (Figure 4E). Then we detected the key proteins, including P62, Keap1, Nrf2, HMOX1, and NQO1 (Figures 4F,G). Western blot analysis revealed a decrease in Keap1 expression and an increase in P62, Nrf2, HMOX1, and NQO1 levels in HGC27 and AGS cells. These findings suggest that P62 expression increased with higher PAH concentrations, leading to its interaction with Keap1. This interaction sequestered Keap1 into P62-mediated protein aggregates (Wang et al., 2021), inhibiting its ability to ubiquitinate Nrf2 and thereby preventing Nrf2 degradation. Consequently, Nrf2 remained stable and accumulated in the cell, enhancing its binding to ARE and activating the antioxidant pathway to maintain redox homeostasis. In summary, PAH not only promotes nuclear translocation of Nrf2 in gastric cancer cells but also concurrently induces oxidative stress while activating the P62-Keap1-Nrf2 pathway.

### 3.4 PAH sensitizes gastric cancer cells to ferroptosis

**FIGURE 5:**
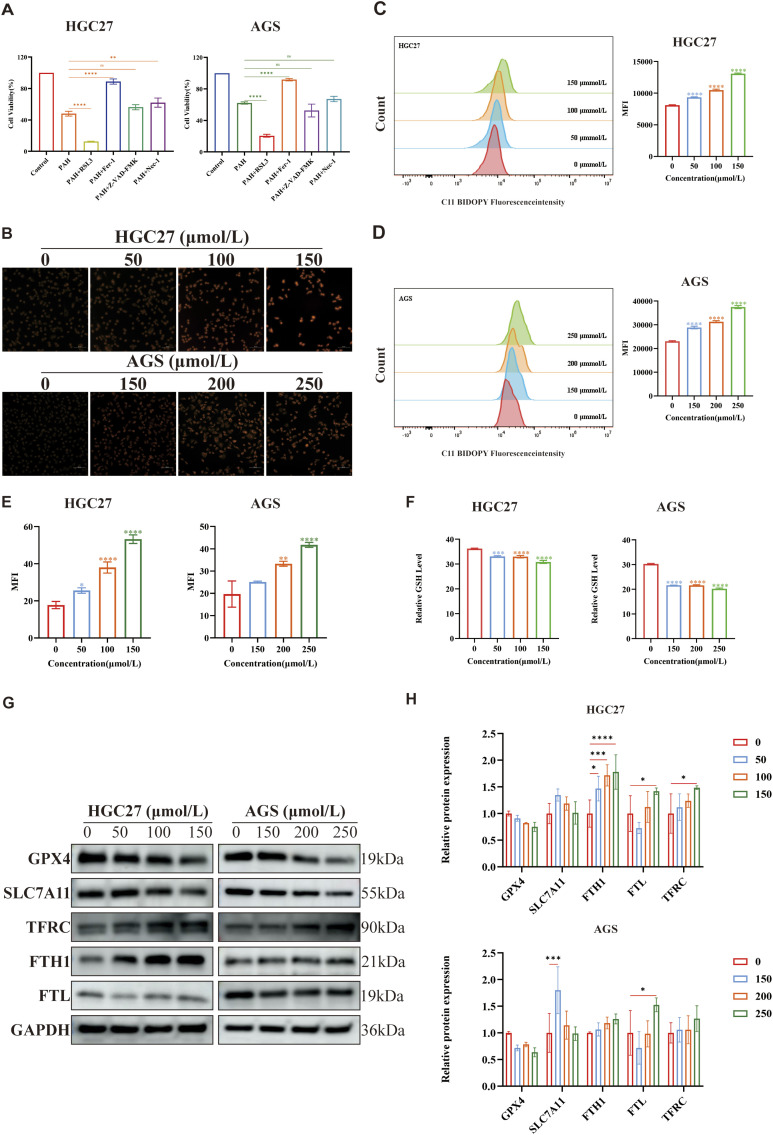
PAH sensitizes gastric cancer cells to ferroptosis **(A)** After treating HGC27 and AGS cells with Z-VAD-FMK, Nec-1, RSL3 and Fer-1 combined with PAH for 24 h, Cell viability was detected by MTT assay (*n* = 4). **(B,E)** FerroOrange fluorescent staining was used to evaluate intracellular Fe^2+^ levels in HGC27 and AGS cells after PAH treatment, images were captured using an inverted fluorescence microscope, and then semi-quantitatively analyzed their mean fluorescence intensity (MFI) using ImageJ software (*n* = 4). Scale bar: 50 μm. **(C,D)** Flow cytometry was used to measure the fluorescence intensity of lipid peroxide indicator BODIPY-581/591 C11 to detect lipid peroxide levels in HGC27 and AGS cells after PAH treatment for 24 h (*n* = 3). **(F)** After HGC27 and AGS cells were treated with PAH for 24 h, intracellular GSH levels were detected (*n* = 3). **(G,H)** Western blotting was used to detect the protein expression of GPX4, SLC7A11, TFRC, FTH1 and FTL (*n* = 3), and ImageJ software was used to quantify the protein bands (Data are expressed as mean ± SD; *p < 0.05, **p < 0.01, ***p < 0.001, ****p < 0.0001, significantly different from the control group).

Ferroptosis is an iron-dependent form of regulated cell death (Tang et al., 2019) characterized by the accumulation of iron-dependent lipid ROS (Dixon et al., 2012), leading to elevated intracellular oxidative stress and iron overload. Transcriptome sequencing revealed that the death of PAH-treated gastric cancer cells is linked to ferroptosis (Figure 4C). To confirm whether the cell death is specifically due to ferroptosis and to exclude other cell death pathways, we employed specific inhibitors of apoptosis, necroptosis, and ferroptosis. HGC27 and AGS cells were treated with PAH in combination with the ferroptosis inhibitor Ferrostatin-1 (Fer-1; 2 μmol/L), the apoptosis inhibitor Z-VAD-FMK (20 μmol/L), and the necroptosis inhibitor Necrostatin-1 (Nec-1; 20 μmol/L) (Figure 5A). The results demonstrated that neither the apoptosis inhibitor Z-VAD-FMK nor the necroptosis inhibitor Nec-1 mitigated the cytotoxic effects of PAH, indicating that ferroptosis is the primary mechanism through which PAH induces cell death in HGC27 and AGS cells.

The system Xc(−)/GSH/GPX4 axis represents the classic regulatory pathway of GPX4 in the mechanism of ferroptosis, where inhibition of the xCT system indirectly depletes intracellular GSH, thereby suppressing GPX4 activity and ultimately leading to ferroptosis (Zhang et al., 2022). To investigate whether PAH-induced ferroptosis is associated with this axis, we employed RSL3, a specific inhibitor of GPX4 known to induce ferroptosis (Chen et al., 2021). The MTT assay results demonstrated that RSL3 significantly enhanced PAH’s ability to induce ferroptosis in cells (Figure 5A). Furthermore, these findings indicate that PAH can synergize with RSL3 to promote ferroptosis.

Increased intracellular Fe^2+^ levels, elevated lipid peroxidation, and GSH depletion are key hallmarks of ferroptosis. Following PAH treatment in HGC27 and AGS cells, we observed a rise in intracellular Fe^2+^ and lipid peroxidation alongside a reduction in GSH levels (Figures 5B–F). In addition, Western blot analysis has demonstrated that PAH treatment downregulated the expression of GPX4 and SLC7A11, while upregulating the expression of transferrin receptor (TFRC), ferritin heavy chain (FTH1), and ferritin light chain (FTL), which are associated with intracellular Fe^2+^ regulation (Figures 5G,H). Ferritin, a highly conserved protein composed of FTH1 and FTL, plays a pivotal role in iron metabolism: FTH1 catalyzes the oxidation of Fe^2+^, while FTL facilitates Fe^3+^ storage. Studies have demonstrated that oxidative stress response proteins, including Nrf2, can activate FTH1 and FTL in cancer cells (Shesh and Connor, 2023), reducing free iron in the labile iron pool (LIP) and inhibiting ferroptosis (Tian et al., 2020; Fang et al., 2021). Consistent with this, we found that PAH treatment increased Nrf2 expression, which in turn activated ferritin expression, resulting in elevated levels of FTH1 and FTL as a protective response against ferroptosis.

### 3.5 Combined use modulates PAH efficacy in treating gastric cancer

**FIGURE 6:**
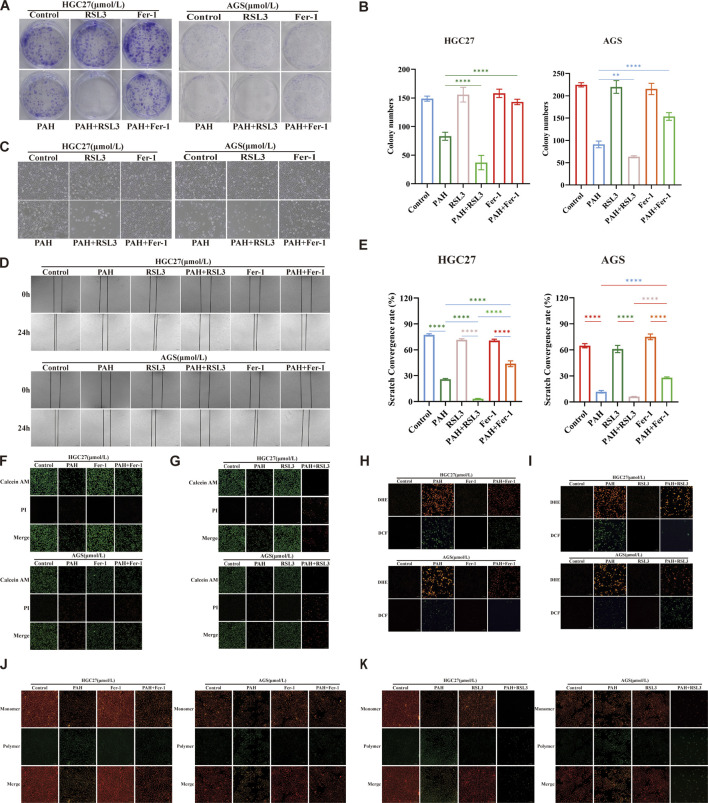
Combination therapy regulates PAH-induced oxidative stress **(A,B)** Colonies of HGC27 and AGS cells after 24 h of treatment with PAH combined with Fer-1 or PAH combined with RSL3, and quantification of colony formation results using ImageJ software (*n* = 4). **(C)** Cell morphology of HGC27 and AGS cells after 24 h of combined treatment. Scale bar, 100 μm. **(D,E)** Scratch images taken under a microscope after 24 h of combined treatment and cell migration analyzed using ImageJ software (*n* = 4). Scale bar, 100 μm. **(F,G)** Calcein AM/PI fluorescence staining to detect cell viability and cytotoxicity after combined treatment (*n* = 4), scale bar: 100 μm. **(H,I)** Dihydroethidium (DHE) and H_2_DCFDA (DCF) fluorescence staining to evaluate the changes in ROS in HGC27 and AGS cells after combined treatment, scale bar: 100 μm. **(J,K)** JC-1 staining to detect changes in membrane potential of HGC27 and AGS cells after combined treatment (*n* = 4), scale bar: 100 μm (Data are expressed as mean ± SD, *n* = 4. ns: no statistical significance, * indicates compared with the control group, *p < 0.05, **p < 0.01, ***p < 0.001, ****p < 0.0001).

We conducted experiments combining PAH with the ferroptosis inhibitor Fer-1 or the ferroptosis inducer RSL3 and evaluated its effect through colony formation experiments (Figure 6A). The colony formation rate was quantified using ImageJ software (Figure 6B). The results indicated that when making a comparison with the PAH treatment group alone, the combination of PAH and RSL3 significantly inhibited the proliferation of cells. In contrast, the combination of PAH and Fer-1 partially restored the proliferation capacity of these cells. Microscopic observation of HGC27 and AGS cell morphology (Figure 6C) revealed similar phenomenon. These findings were also further corroborated by the results of Calcein-AM/PI fluorescence staining (Figures 6F,G). We employed a cell scratch assay. The combination of PAH and Fer-1 restored the migration ability of HGC27 and AGS cells, whereas the combination of PAH and RSL3 inhibited their migratory capacity (Figures 6D,E). We also evaluated oxidative stress levels following the combined treatments. DCF/DHE staining revealed that the combination of PAH and Fer-1 reduced intracellular oxidative stress compared to PAH treatment alone, while the combination of PAH and RSL3 enhanced PAH’s ability to induce oxidative stress (Figures 6H,I). Similarly, JC-1 staining results indicated that the combination of PAH and Fer-1 restored MMP and improved mitochondrial function, whereas the combination of PAH and RSL3 exacerbated mitochondrial damage (Figures 6J,K).

**FIGURE 7:**
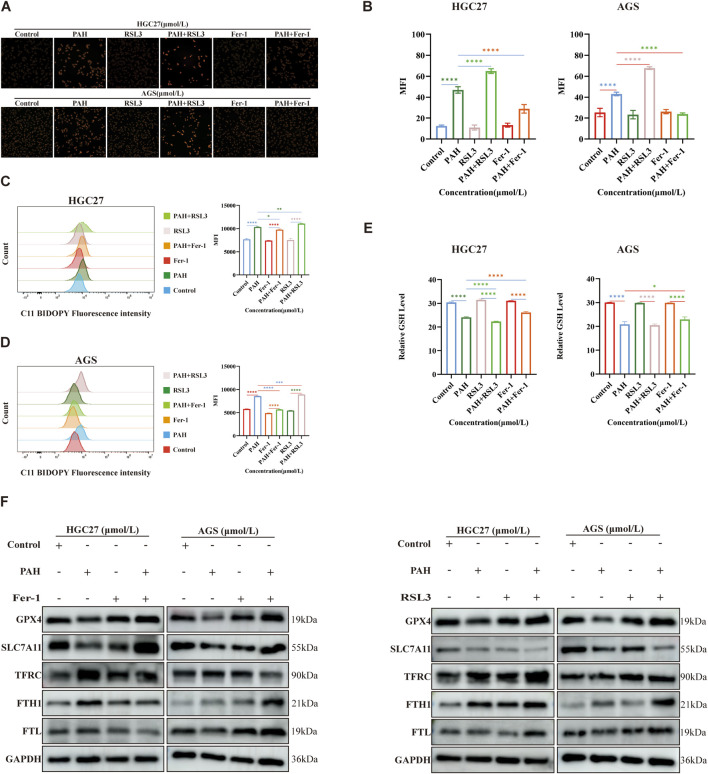
Combined use enhances the efficacy of PAH in regulating ferroptosis **(A–E)** After treatment with PAH combined with Fer-1 or PAH combined with RSL3 for 24 h, FerroOrange fluorescent staining was used to detect Fe^2+^ in HGC27 and AGS cells, flow cytometry was used to detect lipid peroxidation, and the GSH content was detected with a kit and quantified (*n* = 4), scale bar: 50 μm. **(F)** Western blotting detects the expression of ferroptosis-related proteins (GPX4, SLC7A11, TFRC, FTH1, FTL) (Data are expressed as mean ± SD; *p < 0.05, **p < 0.01, ***p < 0.001, ****p < 0.0001, significantly different from the control group).

We next re-evaluated the changes in intracellular ferroptosis-related indicators following the combined treatment of PAH with Fer-1 and PAH with RSL3. Specifically, we measured the levels of Fe^2+^, lipid peroxidation, and GSH in HGC27 and AGS cells (Figures 7A–E). The combination of PAH and Fer-1 reduced the levels of intracellular Fe^2+^ and lipid peroxidation levels, alongside an increase in intracellular GSH levels. In contrast, the combination of PAH and RSL3 yielded the opposite effect. Compared to treatment with PAH alone, the combined use of PAH and RSL3 further intensified the degree of cell ferroptosis.

We conducted Western blotting to assess changes in intracellular ferroptosis-related protein levels following combination treatments (Figure 7F). Compared to the PAH-only treatment group, the combined use of PAH and Fer-1 in HGC27 cells resulted in increased protein expression of GPX4 and SLC7A11. Besides, the expression of TFRC was reduced, while FTH1 and FTL were also decreased. FTH1 catalyzes the oxidation of Fe^2+^ produced during intracellular ferroptosis, and FTL stores the resulting Fe^3+^ after oxidation. Both proteins are significantly consumed, thereby inhibiting the accumulation of intracellular Fe^2+^. In AGS cells, the combined use of PAH and Fer-1 led to increased protein expression of GPX4 and SLC7A11, decreased expression of TFRC, and elevated levels of FTH1 and FTL. The increased FTH1 and FTL likely contributed to the inhibition of further ferroptosis in these cells. Conversely, when treated with a combination of PAH and RSL3, in HGC27 cells, the expression of TFRC was increased and SLC7A11 was decreased. Interestingly, the protein levels of GPX4, FTH1, and FTL were also elevated; In AGS cells, the expression of SLC7A11 was further reduced, while TFRC, GPX4, FTH1, and FTL were increased. We hypothesize that the upregulation of GPX4 may represent a cellular response to counteract the increase in ROS caused by enhanced ferroptosis over a short period. Similarly, the increased expression of FTH1 and FTL may reflect a compensatory mechanism triggered by the sharp rise in intracellular Fe^2+^. However, it is urgent for further research to elucidate the underlying mechanisms driving these observations.

### 3.6 PAH regulates ferroptosis through the P62-Keap1-Nrf2 pathway

**FIGURE 8:**
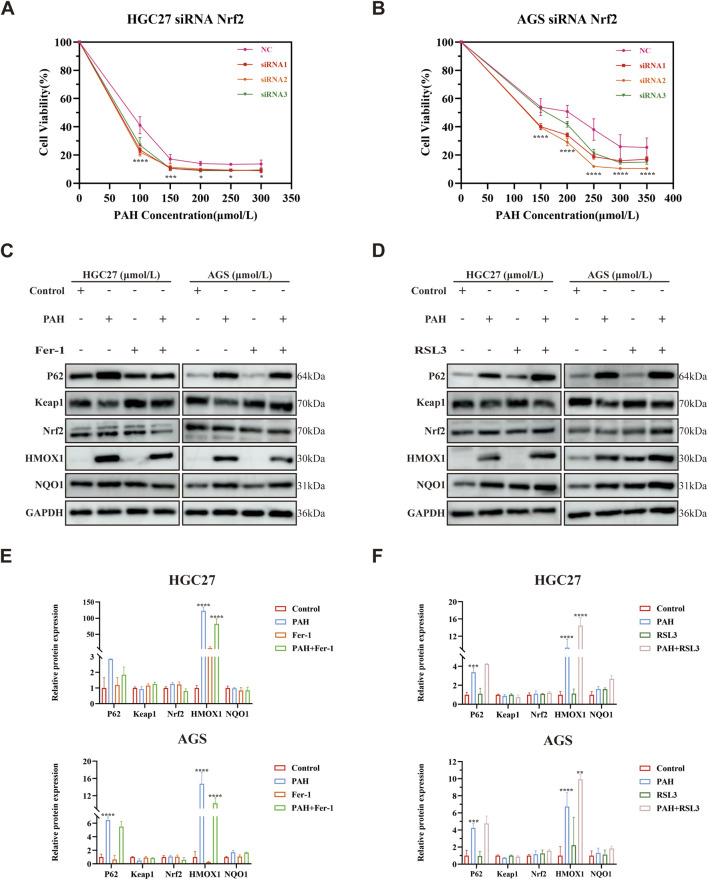
PAH regulates ferroptosis through the P62-Keap1-Nrf2 pathway. **(A,B)** Cell viability was assessed by MTT assay after 24-h treatment with PAH and (or) siRNA Nrf2 in HGC27 and AGS cells (*n* = 4). **(C)** Western blot assay of P62, Keap1, Nrf2, HMOX1 and NQO1 protein expression in HGC27 and AGS cells after 24 h of PAH and Fer-1 co-treatment (*n* = 3). **(D)** Western blot assay of P62, Keap1, Nrf2, HMOX1 and NQO1 protein expression in HGC27 and AGS cells after 24 h of PAH and RSL3 co-treatment (*n* = 3). **(E)** ImageJ software was used to quantify the protein bands in HGC27 and AGS cells after 24 h of PAH and Fer-1 co-treatment (*n* = 3). **(F)** ImageJ software was used to quantify the protein bands in HGC27 and AGS cells after 24 h of PAH and RSL3 co-treatment (*n* = 3) (Data are expressed as mean ± SD; *p < 0.05, **p < 0.01, ***p < 0.001, ****p < 0.0001, significantly different from the Control or PAH group).

To investigate whether PAH regulates ferroptosis through the P62-Keap1-Nrf2 pathway and for a more comprehensive understanding of Nrf2’s functional role in regulating ferroptosis in this model, we performed Nrf2 knockdown experiments using siRNA transfection in HGC27 and AGS cells. Following 24-h treatment with corresponding concentrations of PAH, cell viability was assessed. Notably, all three siRNA Nrf2 target sequences could enhance PAH-induced ferroptosis compared to PAH treatment alone (Figures 8A,B). Meanwhile, we examined the protein expression levels of the P62-Keap1-Nrf2 pathway and its downstream target genes, HMOX1 and NQO1, following combination treatment with PAH and Fer-1 or PAH and RSL3. Western blot analysis revealed the following findings (Figures 8C–F): After 24 h of co-treatment with PAH and Fer-1, the expression of P62 decreased compared to the group treated with PAH alone. Besides, Nrf2 expression was further decreased, and the protein expression of its downstream target genes, HMOX1 and NQO1, were also decreased, indicating that the inhibition of intracellular ferroptosis partially restored cell viability, weakened the activation of the antioxidant system. In contrast, after 24 h of co-treatment with PAH and RSL3, compared to the PAH-only group, P62 expression increased and Nrf2 expression was elevated. Consequently, the expression of HMOX1 and NQO1 also increased. These findings indicate that the combined action of PAH and RSL3 exacerbated ferroptosis, leading to heightened oxidative stress levels within the cells. In response, the P62-Keap1-Nrf2 pathway was activated to counteract the increased ROS levels resulting from the intensification of ferroptosis, consequently suppressing the progression of ferroptosis in gastric cancer cells.

## 4 Discussion

Gastric cancer still ranks as one of the main reasons for cancer-related mortality, primarily due to its asymptomatic nature in early stages, non-specific clinical manifestations in later stages, and inherent biological and genetic heterogeneity. So, these factors contribute to low rates of early diagnosis and poor overall prognosis (Tan and Yeoh, 2015). Current therapeutic strategies for gastric cancer encompass surgical intervention, cytotoxic therapies, targeted therapy, immunotherapy, biomarker-guided therapy, and intestinal microbiota-based treatments (Sexton et al., 2020; Guan et al., 2023). Despite the availability of these diverse treatment modalities, most cases of gastric cancer are diagnosed at an advanced stage, significantly limiting the clinical applicability and effectiveness of surgical options (Durães et al., 2014). While conventional systemic chemotherapy has markedly improved outcomes for patients with advanced gastric cancer, challenges such as drug resistance, toxicity, and patient intolerance persist. Statistics indicate that approximately 60% of gastric cancer patients are unable to undergo curative treatment due to late-stage diagnosis or the presence of comorbidities (Allum et al., 2018). Targeting the epidermal growth factor receptor (EGFR) also represents a primary approach in gastric cancer therapy. Yet, aberrant RNA m5C methylation contributes to intrinsic resistance against anti-EGFR treatments (Wang Y. et al., 2023), driving disease aggressiveness and constraining the development of effective targeted therapies. Furthermore, even with multimodal treatment approaches, the recurrence of gastric cancer remains a common issue (Joshi and Badgwell, 2021). Given these challenges, it is an urgent need that the development of novel therapeutic strategies should be carried out to improve the prognosis and outcomes for patients with gastric cancer.

Natural products derived from plants, animals, and microorganisms serve as a major source of therapeutic agents for treating human cancer. These natural compounds represent a valuable reservoir for the development and exploration of novel anti-tumor drugs. Natural products exhibit a broad spectrum of anti-tumor properties, offering significant advantages such as multi-target efficacy, low toxicity, structural stability, reduced risk, and economic viability (Deng et al., 2024). Therefore, natural products have huge potential for application in targeted gastric cancer therapy. In this study, we focused on perillaldehyde, a major component of essential oil derived from the perilla plant, which is widely used both as a leafy vegetable and in traditional medicine (Erhunmwunsee et al., 2022). We investigated its therapeutic potential against gastric cancer. Our findings showed that PAH could significantly induce the death of gastric cancer cells *in vitro*. Moreover, the proliferation or migration of gastric cancer was effectively inhibited by PAH. To further validate its efficacy, we established a subcutaneous tumor model using MFC cells in BALB/c nude mice, demonstrating PAH’s ability to suppress gastric cancer growth *in vivo*. Moreover, PAH exhibited milder toxic side effects and demonstrated a favorable safety profile for major tissues and organs.

It is evident that the regulation of oxidative stress is a pivotal element in the progression of tumors and the response to anticancer therapies (Gorrini et al., 2013). A multitude of signaling pathways linked to tumor development have the capacity to regulate the metabolism of ROS through both direct and indirect mechanisms (Gorrini et al., 2013). A considerable body of research has indicated that elevated levels of ROS can play a pivotal role in the promotion of oncogenic phenotypes and inhibit tumor progression by enhancing sensitivity to cell death (Cheung and Vousden, 2022). Nrf2 is a major regulator of cellular antioxidant responses, its negative regulator Keap1 forms the classical pathway for Nrf2 regulation (Rojo de la Vega et al., 2018). Moreover, a non-classical regulatory pathway exists, which is Keap1-dependent and involves the autophagy-related protein P62/SQSTM1 (Komatsu et al., 2010; Lau et al., 2010). Together, these components form the P62-Keap1-Nrf2 pathway, which modulates oxidative stress levels in tumor cells. In our study, we employed the JC-1 mitochondrial membrane potential assay, which revealed that PAH treatment reduced the MMP in HGC27 and AGS gastric cancer cells, concurrently inducing ROS generation. This finding was further supported by DCF and DHE staining assays. Immunofluorescence analysis revealed that PAH treatment activated Nrf2 expression and promoted its nuclear translocation in both HGC27 and AGS cell lines. Western blot analysis demonstrated that PAH treatment led to an upregulation of P62 expression and a downregulation of Keap1. Under physiological conditions, Nrf2 is maintained at low basal levels through Keap1-mediated proteasomal degradation. However, PAH treatment significantly downregulates Keap1 expression, thereby inhibiting constitutive Nrf2 degradation. This stabilization facilitates Nrf2 accumulation in the cytoplasm, followed by nuclear translocation, ultimately leading to transcriptional activation of downstream antioxidant genes and subsequent protein expression (Baird and Yamamoto, 2020). In fact, we indeed observed elevated expression levels of HMOX1 and NQO1. Early studies on oxidative stress response regulation revealed that *NRF2*-encoding transcripts are not upregulated in response to oxidative stress, indicating that Nrf2 activity is primarily regulated through post-transcriptional mechanisms (Baird and Yamamoto, 2020). Thus, Nrf2 activation typically occurs as a secondary response to oxidative stress. While PAH treatment activated Nrf2 protein expression in gastric cancer cells, transcriptome sequencing data failed to detect corresponding *NRF2* mRNA upregulation (Supplementary Material). This suggests that Nrf2 activation likely represents a compensatory response to PAH-induced oxidative stress rather than direct transcriptional regulation by PAH (Baird et al., 2013).

Iron overload is frequently associated with oxidative stress (Chen et al., 2022), and the excessive accumulation of iron can lead to ferroptosis. Nrf2, a key transcription factor, plays a critical role in modulating intracellular iron levels by transcriptionally activating downstream genes involved in iron metabolism (Jiang et al., 2024). Besides, Nrf2 regulates ferroptosis in tumor cells by inducing the expression of NQO1, HMOX1, and FTH1 (Sun et al., 2016). In this study, we hypothesized that PAH induces ferroptosis while simultaneously promoting oxidative stress. To investigate this, we conducted RNA sequencing on AGS cells treated with PAH for 24 h. KEGG functional enrichment analysis and Reactome pathway enrichment analysis revealed that PAH could promote oxidative stress and cell death through mechanisms involving not only the P62-Keap1-Nrf2 pathway but also glutathione metabolism, fatty acid metabolism, and ferroptosis. These findings aligned with our initial hypothesis. Subsequent experimental validation confirmed that PAH indeed induces ferroptosis. Western blotting revealed that PAH treatment significantly decreased intracellular levels of GPX4 and SLC7A11, while increasing protein expression of transferrin receptor (TFRC), ferritin heavy chain (FTH1), and ferritin light chain (FTL). Previous studies have established GPX4 and SLC7A11 as downstream targets of Nrf2, where Nrf2 activation leads to GPX4 overexpression in AML, contributing to chemoresistance and ferroptosis evasion (Liu et al., 2023). However, in our study, while PAH activated Nrf2 expression in both HGC27 and AGS cells, it paradoxically reduced GPX4 and SLC7A11 levels, showing an inverse correlation with Nrf2 activation. We propose this represents a context-dependent effect of PAH treatment. Although PAH-induced Nrf2 activation serves as a compensatory response to ferroptosis-mediated oxidative stress and may transcriptionally upregulate GPX4, the protein could undergo degradation due to persistent lipid peroxide accumulation (Yang et al., 2016). Meanwhile, the levels of activating transcription factor 4 (ATF4) are positively correlated with those of SLC7A11. ATF4 and Nrf2 coordinately regulate the expression of SLC7A11 (He et al., 2023). Our transcriptome sequencing results showed reduced transcriptional activity of *ATF4* (Supplementary Material). Therefore, we hypothesize that: on one hand, under conditions of persistent ferroptosis activation, the expression of SLC7A11 may be negatively regulated by ATF4, thereby antagonizing the transcriptional activation effect of Nrf2 on it; on the other hand, with the increase in concentration, PAH-induced ferroptosis gradually enhances the inhibition of SLC7A11 protein expression, leading to a decrease in intracellular SLC7A11.

We further explored the effects of PAH in combination with Fer-1 and RSL3. The results showed that PAH could regulate the efficacy of ferroptosis and affect the expression of SLC7A11, GPX4, FTH1, and FTL. Notably, when PAH was combined with RSL3, it significantly enhanced RSL3’s ability to induce ferroptosis. This observation suggests that natural products, such as PAH, could serve as synergistic agents in ferroptosis-based therapeutic strategies for gastric cancer. We investigated the role of the P62-Keap1-Nrf2 pathway, and our findings indicate that PAH regulates ferroptosis through this pathway, establishing a clear regulatory relationship between the P62-Keap1-Nrf2 signaling axis and PAH-induced ferroptosis.

The combination of natural products with clinically used chemotherapeutic or targeted drugs represents a promising synergistic strategy for gastric cancer treatment with significant translational potential. Our previous studies have demonstrated that the natural compound Cepharanthine (CEP) can enhance the anti-tumor effects of doxorubicin (DOX) and cisplatin (CIS) in gastric cancer (Lu et al., 2024). Therefore, to investigate whether PAH exhibits synergistic effects when combined with chemotherapeutic drugs DOX and CIS, we evaluated the efficacy of PAH in combination with DOX or CIS using the MTT assay in HGC27 and AGS cells. The results showed that monotherapies with PAH, DOX, or CIS alone reduced cell viability, whereas combination treatments significantly decreased cell viability (Supplementary Figures S1A–D). Our preliminary findings indicate that PAH synergizes with conventional chemotherapeutic drugs DOX or CIS in gastric cancer, exerting a combined anticancer effect. However, the specific mechanisms underlying cell death induced by the combination of PAH and chemotherapeutic drugs remain unclear and warrant further investigation.

This study offers novel insights into the mechanisms by which PAH inhibits gastric cancer growth and provides a theoretical and experimental foundation for its potential as both a ferroptosis inducer and a regulator of the P62-Keap1-Nrf2 pathway. These findings suggest promising possibilities for the development of PAH as a therapeutic agent for gastric cancer, particularly in combination with existing clinical treatments, to achieve a more robust anti-tumor effect. However, the safety and efficacy of PAH in human patients continue to be fully elucidated, necessitating further research to evaluate its potential applications in cancer therapy.

## 5 Conclusions

In summary, this study (Supplementary Figure S2) revealed that PAH inhibits gastric cancer growth both *in vitro* and *in vivo* via inducing oxidative stress. Importantly, PAH maintains intracellular redox homeostasis by counteracting oxidative stress through the activation of the P62-Keap1-Nrf2 pathway. PAH sensitizes gastric cancer cells to ferroptosis, it regulates ferroptosis by activating the P62-Keap1-Nrf2 antioxidant system. Furthermore, PAH synergizes with ferroptosis inducer RSL3 to promote ferroptosis in gastric cancer. These findings provide further insight into the potential anti-tumor mechanisms of PAH, highlighting PAH-induced ferroptosis as a promising therapeutic strategy for the treatment of gastric cancer. Meanwhile, the combination of the natural product PAH with chemotherapeutic drugs clinically used for gastric cancer holds promise as a treatment strategy with translational impact.

## Data Availability

The datasets presented in this study can be found in online repositories. The names of the repository/repositories and accession number(s) can be found in the article/Supplementary Material.

## Abbreviations

PAH, Perillaldehyde; ROS, Reactive oxygen species; System Xc (−), The cystine/glutamate antiporter system; BSA, bovine serum albumin; GSH, Glutathione; P62/SQSTM1, Sequestosome-1; Keap1, Kelch-like ECH-associated protein 1; NFE2L2/Nrf2, Nuclear factor erythroid 2-related factor 2; GPX4, Glutathione peroxidase 4; TRIM21, Tripartite motif-containing protein 21; SLC7A11/xCT, Solute Carrier Family 7, Member 11; FTH1, Ferritin heavy chain 1; FTL, Ferritin light chain; GAPDH, Glyceraldehyde 3-phosphate dehydrogenase; TFRC, Transferring receptor; NQO1, NAD(P)H quinone dehydrogenase 1; HO-1, Heme oxygenase 1; FBS, Fetal bovine serum; MTT, 3-(4,5-Dimethylthiazol-2-yl)-2,5-diphenyltetrazolium bromide; 5-FU, 5-Fluorouracil; DOX, Doxorubicin; CIS, Cisplatin; PBS, Phosphate buffered saline; DHE, Dihydroethidium; DCF, H_2_DCFDA; PMSF, Phenylmethanesulfonylfluoride; BCA, Bicinchoninic acid; PVDF, Polyvinylidene difluoride; HRP, Horseradish peroxidase; ECL, Enhanced chemiluminescence; IC50, Half maximal inhibitory concentration; MMP, Mitochondrial membrane potential; KEGG, Kyoto Encyclopedia of Genes and Genomes; LIP, Labile iron pool; EGFR, epidermal growth factor receptor; ATF4, activating transcription factor 4.
