# Supplementary figures and images for: Perillaldehyde synergizes with ferroptosis inducers to promote ferroptotic cell death in gastric cancer
Source: Front Cell Dev Biol. 2025 Jun 3;13:1598520. doi: 10.3389/fcell.2025.1598520 (PMC12170665; doi:10.3389/fcell.2025.1598520)

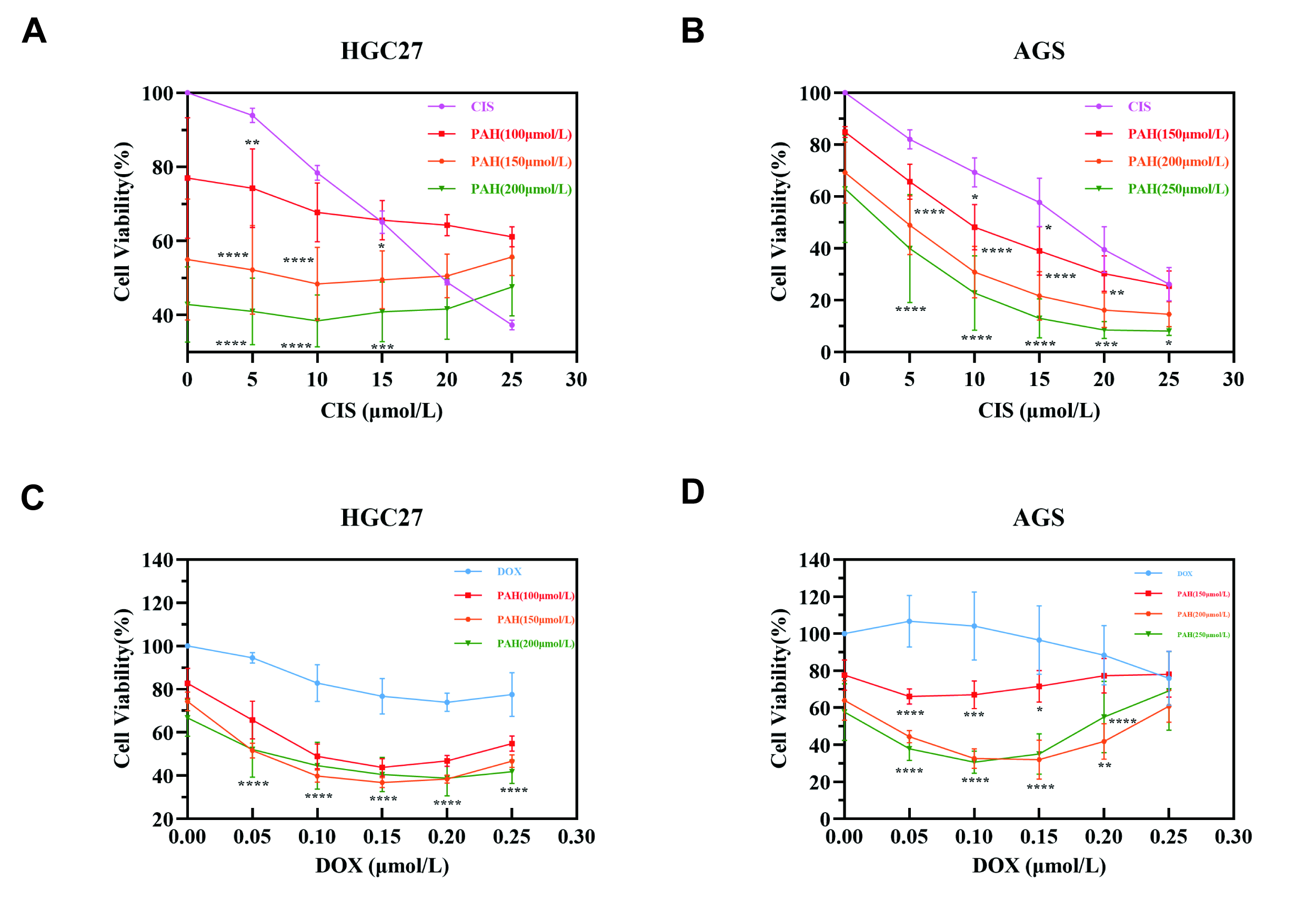

Supplement: Supplementary file 2 [file Image1.tif]
